# Non-Contact Vital Signs Monitoring of Dog and Cat Using a UWB Radar

**DOI:** 10.3390/ani10020205

**Published:** 2020-01-25

**Authors:** Pengfei Wang, Yangyang Ma, Fulai Liang, Yang Zhang, Xiao Yu, Zhao Li, Qiang An, Hao Lv, Jianqi Wang

**Affiliations:** Department of Medical Electronics, School of Biomedical Engineering, The Fourth Military Medical University, Xi’an 710032, China; wangpf2016@fmmu.edu.cn (P.W.); mayangyang@fmmu.edu.cn (Y.M.); liangfulai@fmmu.edu.cn (F.L.); yangzhang@fmmu.edu.cn (Y.Z.); yuxiao@fmmu.edu.cn (X.Y.); lizhaofmmu@fmmu.edu.cn (Z.L.); qiang.an.903@outlook.com (Q.A.)

**Keywords:** cat, dog, vital signs monitoring, radar, ultra-wideband (UWB), variational mode decomposition (VMD)

## Abstract

**Simple Summary:**

In this paper, we presented a new non-contact method to measure the vital signs of at-rest pets, where the ultra-wideband radar was applied for previously unachievable sensing ability and testing convenience. The proposed scheme did not interfere with the daily rhythm of the pet being tested; most subjects would not even notice the ongoing real-time monitoring due to the larger measurement coverage of the radar sensor. Through this work, we provided an innovative tool to promote not only the measurement of pet vital signs but also the improvement of animal welfare to prevent invasive, dangerous, and unacceptable contact measurement techniques, such as anesthesia, hair removal, and surgical implants. In addition, the proposed method could realize the daily vital signs measurement and sleep monitoring of dogs and cats, which is important for the diagnosis and timely treatment of pet diseases.

**Abstract:**

As pets are considered members of the family, their health has received widespread attention. Since pets cannot talk and complain when they feel uncomfortable, monitoring vital signs becomes very helpful in disease detection, as well as observing their progression and response to treatment. In this study, we proposed an ultra-wideband radar-based, non-contact animal vital sign monitoring scheme that could monitor the breathing and heart rate of a pet in real-time. The primary advantage of the ultra-wideband radar was its ability to operate remotely without electrodes or wires and through any clothing or fur. Because of the existing noise and clutter in non-contact detection, background noise removal was applied. Furthermore, the respiration rate was directly obtained through spectrum analysis, while the heartbeat signal was extracted by the variational mode decomposition algorithm. By using electrocardiogram measurements, we verified the accuracy of the radar technology in detecting the anesthetized animals’ respiratory rate and heart rate. Besides, three beagles and five cats in a non-sedated state were measured by radar and contact pressure sensors simultaneously; the experimental results showed that radar could effectively measure the respiration of cats and dogs, and the accuracy rate was over 95%. Due to its excellent performance, the proposed method has the potential to become a new choice in application scenarios, such as pet sleep monitoring and health assessment.

## 1. Introduction

As the most popular pets in society, cats and dogs are often regarded as family members, and as such, their health has attracted widespread attention. Since pets are unable to talk and complain when feeling uncomfortable, monitoring vital signs, including breathing and heartbeat, becomes very helpful in detecting diseases, as well as observing their behavior and response to treatment [1].

Respiration and heart rates in dogs and cats are the most critical objective parameters of the physical examination. A significant increase or decrease in the respiration or heart rate of the dog may be a sign of a major illness, such as dehydration, heart disease, fever, or shock. Besides, heart rate is also commonly used as an emotion-related physiological indicator for assessing the mental state of cats and dogs, such as anxiety and depression [2,3,4,5].

Currently, the contact sensor is a common solution for measuring vital signs of animals with body coverings, which often involves complex and invasive preparation procedures while causing significant distress to the animals [6]. Sometimes, they would be anesthetized or severely restricted to prevent movement that can damage the measurement setup [7,8]. For example, an electrocardiogram (ECG), which is often used for heartbeat waveforms, requires stable electrical contact of the skin-electrode, requiring the pet hair to be removed during measurement, and in some cases, even anesthesia is needed [9,10,11,12]. Meanwhile, a collar for pet animals is also another popular measurement method, which measures the vital signs through sensors that come into contact with the body of the animal [13,14]. However, if one needs to utilize a susceptible sensor on the neck to capture such signals, one would have to make the collar very tight around the neck of the pet, which could negatively affect the behavior and comfort of the animal. On the other hand, auscultation and ultrasound are also commonly used measurement methods. However, they require close skin contact or impedance matching gels to obtain a clear signal, thus requiring a high degree of animal handling and a wealth of experience [15,16,17,18,19].

Compared to contact sensors, non-contact vital sign detection does not require any sensor attached to the target body, thus improving target comfort and preventing a change in the physiological parameters of contact-sensitive pets caused by touch. Due to the advantages of non-contact sensors, they have attracted the attention of many researchers. Photoplethysmography (PPG) is a popular optical technology for heart rate monitoring [20,21]. However, due to a short detection range and limited by the condition of the animal body surface, it cannot be applied for monitoring pet vital signs. Similarly, the hair covering the body surface also causes intricacies in the camera or video-based approaches, thus, limiting their application to animals [22,23,24]. Recently, radar, as one of the non-contact vital signs monitoring methods, has received extensive interest and been applied in a variety of scenarios [25,26,27,28,29,30]. In [31,32], a 60-GHz radar was used for the measurement of the cardiorespiratory movement of a laboratory rat. In [33], a millimeter-wave radar was applied to measure the vital signs of a rat and rabbit. To be sufficiently sensitive to the vital signs of small animals, they raised the carrier frequency to the millimeter-wave level, which not only increased the system cost but also reduced the operational distance. In addition, in [34], a radio frequency near-field coherent sensing (NCS) scheme was proposed to monitor small animals in the laboratory. However, the short detection range limits its applicability in monitoring vital signs of pets at home. On the other hand, compared to millimeter-wave radar and NCS, ultra-wideband (UWB) has a large detection range and excellent penetration performance, which facilitates its monitoring at home.

**Figure 1:**
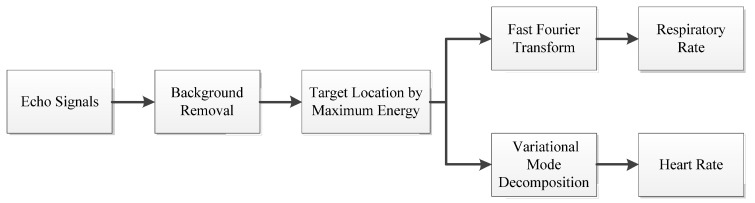
Flow diagram of radar signal processing.

In this study, we proposed a pet vital sign monitoring scheme based on the UWB radar. The flow chart of the proposed scheme is shown in Figure 1. After the target echo signal was received by the radar, the background was removed by subtracting the mean value, while the target was located by calculating the maximum energy at different distances. Furthermore, the respiration rate could be obtained directly by the fast Fourier transform (FFT). Although FFT could convert time-domain signals into frequency domain signals, we still need a better algorithm to obtain weak information, such as the heart rate [35]. Since the variational mode decomposition (VMD) algorithm is efficient and stable in non-stationary signal processing [36], it is widely used in mechanical fault diagnosis [37,38], biomedical signal processing [39,40], and remote sensing signal analysis [41,42]. Therefore, we used it to extract respiratory and heartbeat signals.

As studies involving the non-contact vital sign (breathing and heartbeat) measurement of pets are relatively limited in number, our objective was to provide a low-cost, non-contact, and unconstrained vital signs measurement for cats and dogs, which could significantly promote the welfare of these pets. To verify the performance of our scheme, we used a common measurement method, named electrocardiogram (ECG), and contact pressure sensor to measure the target simultaneously. In addition, to illustrate the practical application value of our approach, we also employed radar to evaluate at-rest animals.

## 2. Materials and Methods

### 2.1. Subjects

Four dogs and five cats participated in the experiment. The four healthy adult beagle dogs (two male and two female) were 1 to 3 years old and weighed 9–13 kg. Respiratory and heartbeat signals were measured for a male beagle under anesthesia and in a conscious state. The vital signs of the other three beagle dogs were measured in a state of rest. The five healthy cats (two male and three female) were 1.5 to 3 years old and weighed 2–5 kg, including a Ragdoll cat, a British shorthair cat, and three ordinary DragonLi cats. Similar to the dogs, these cats were also measured in a state of rest.

All experimental animals were provided by the Fourth Military Medical University. The Experimental Animal Welfare and Ethics Committee of the Fourth Military Medical University approved all the experimental procedures (No. 20190901).

### 2.2. UWB Radar System

For pet vital sign monitoring, a typical UWB radar system on chip (SoC) named X4M02 (XeThru/Novelda, Oslo, Norway) was adopted in the experiment. The SoC applied in this study featured a transmitter (TX) that complied with regulations for unlicensed operation, direct-radio frequency (RF) sampling using the swept-threshold (ST) technique, and RF interference rejection.

**Figure 2:**
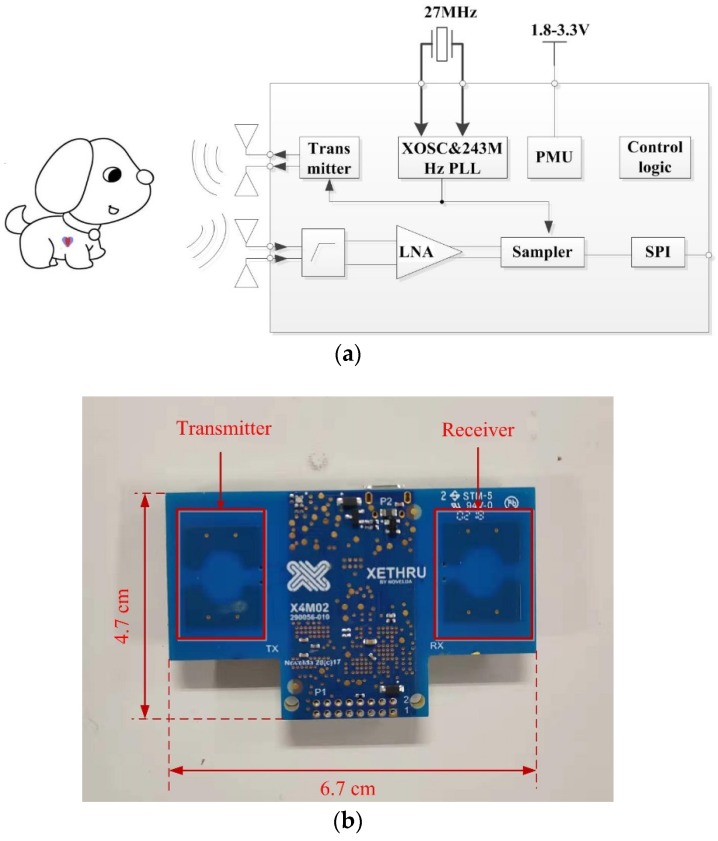
(**a**) Block diagram of the radar sensor SoC (system on chip); (**b**) the image of a real radar sensor.

As shown in Figure 2a, there existed many components in the system architecture. The TX transmitted pulses with an interval determined by the pulse repetition frequency, while the receiver (RX) received the echo signal after the delay corresponding to the round-trip time of flight (ToF) to the target and return. Meanwhile, based on this echo signal, a frame was created, which digitally represented the time-domain range profile. In addition, the TX-phase-locked loop (PLL) synthesized an accurately controlled pulse. The fully differential RX front comprised a high-pass filter (HPF), a low noise amplifier (LNA), and a sampler. On the other hand, the serial peripheral interface (SPI) was used for communication, while the power management unit (PMU) was used to supply power. The real radar sensor is shown in Figure 2b. The transmitter and receiver were patch antennas and were all integrated on the chip.

The transmitter bandwidth in this study was 1.4 GHz, while the center frequency of the sensor was 7.29 GHz. The receiver sampled the reflected signal at 23.328 GS/s and could cover a 9.9 m consecutive range. Here, we set the detection distance range as 5 m. The SoC was a core controller that communicated with the radar via a USB cable and received raw radar data in real-time. The average output power in dBm/MHz of the center frequency was lower than –44 dBm, which complied with Federal Communications Commission (FCC) and European Telecommunications Standards Institute (ETSI) in terms of average transmitted power.

### 2.3. Data Acquisition

**Figure 3:**
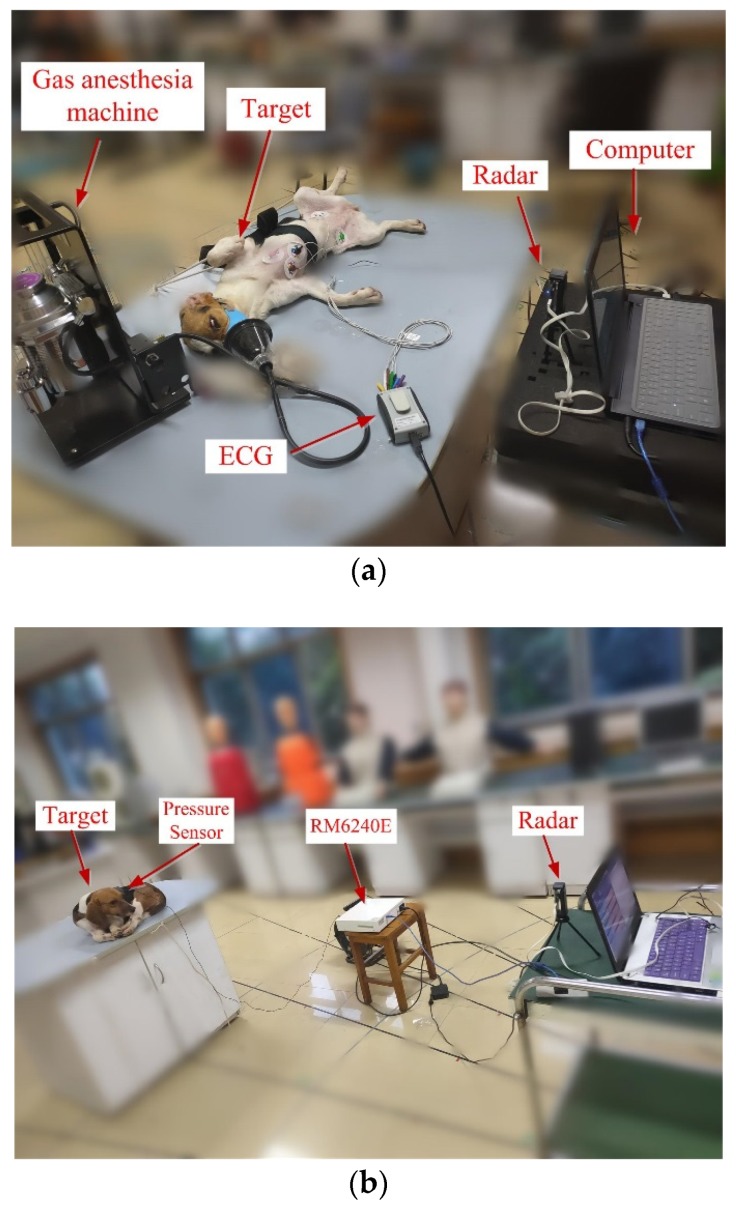
(**a**) Experimental scene when the target is under anesthesia; (**b**) Experimental scene when the target is at rest.

To verify the UWB radar-based measurements of breathing and heart rate of the pets, we first carried out synchronized UWB radar and ECG measurements on a dog. The experimental scene is shown in Figure 3. To reduce the effects of anesthesia on animals, we used gas anesthesia with isoflurane, from which the target could quickly recover. The isoflurane was used by the anesthesia machine. The radar, as shown in the figure, was very small and placed within one meter from the dog. The ECG and radar were connected to the computer for real-time monitoring.

The radar measured the antenna characteristics modulated by the dielectric boundary movement changes, while the ECG measured the body potential differences induced by the minute skin current further induced by the electrical heart stimulation and blood flow. In this perspective, the radar had a waveform transducing the internal and external mechanical motion of the heart, which is related to the mechanical information in ballistocardiogram and seismic cardiography [34,42,43].

Besides, we also used this radar to measure the physiological parameters (respiratory rate and heart rate) of cats and dogs when they were at rest. A typical experimental scenario is shown in Figure 3b, in which, a beagle lies on the experimental table, and about 1 m away from the radar sensor. The physiological signal of the target was acquired synchronously by the pressure sensor, to which a multi-channel physiological signal acquisition system, RM6240E, was connected.

### 2.4. Signal Preprocessing

Echo signals received by the receiving antenna were in the form of a two-dimensional matrix, which was M by N. M is the fast sampling number that represents the detection distance, while N is the slow sampling number that represents the detection time.

For UWB radar systems, when hitting the target, part of the transmitted pulse was reflected due to the high reflectivity of the body. Due to respiration and heartbeat motion, the chest expanded and contracted periodically, causing the distance d(t) to change regularly around the antenna distance, $d_{0}$. For vital signs monitoring, the d(t) could be expressed as:

(1) $$d(t)=d_{0}+m_{b}\sin(2\pi f_{b}t)+m_{h}\sin(2\pi f_{h}t)$$

where $m_{b}$ and $m_{h}$ are the amplitudes, while $f_{b}$ and $f_{h}$ are the frequencies, of respiration and heartbeat.

When multiple channels exist, the received signal could be represented as:

(2) $$r(t,\;\tau )=\sum_{i}A_{i}p(\tau -\tau_{i})+A_{T}p(\tau -\tau_{d}(t))$$

where p(t) is a normalized received pulse, $A_{i}$ is the amplitude of each multipath component, and $\tau_{i}$ is the corresponding delays. $A_{T}$ is the pulse amplitude reflected due to the chest movement. τ is related to the target distance, while t is related to the detection time.

The clutter caused by stationary objects, such as walls and furniture, usually leads to a baseline drift in those signals along the slow time dimension. For removing the clutter of stationary objects, the average of all the values of the waveform was subtracted by a sliding window.

UWB radar sensor itself could measure distance based on the time-of-arrival. Here, we used maximum energy selection because of the high precision measurement of respiratory and heartbeat information. After the above-mentioned preprocessing process, the target could be positioned by calculating the energy along with the distance phase, at which the vital sign signal could be obtained.

### 2.5. Variational Mode Decomposition

The VMD is a new adaptive decomposition method based on Wiener filtering and Hilbert transform [36]. By the optimal solution search of the constrained variational model, the vital signs signal could be decomposed into a set of variational intrinsic mode function (VIMF) components with sparse characteristics. Each mode is compact around a center pulsation $\omega_{k}$, and its bandwidth is estimated using $H^{1}$ Gaussian smoothness of the shifted signal. The VMD is written as a constrained variational problem [36]:

(3) $$\underset{\left\{u_{k}\right\},\left\{\omega_{k}\right\}}{\min}\left\{\sum_{k=1}^{K}{\left\|\partial t\left[\left(\delta (t)+\frac{j}{\pi t})*u_{k}(t\right)\right]e^{-j\omega kt}\right\|}_{2}^{2}\right\}\;s.t.\;\sum_{k=1}^{K}u_{k}=f$$

where K is the number of VIMFs, while f is the input signal; $\left\{u_{k}\right\}=\left\{u_{1},u_{2},\cdots ,u_{K}\right\}$ and $\left\{\omega_{k}\right\}=\left\{\omega_{1},\omega_{2},\cdots ,\omega_{K}\right\}$ are shorthand notations for the set of all modes and their center frequencies, respectively.

Equation (3) could be solved by introducing a quadratic penalty and Lagrangian multipliers. The augmented Lagrangian is given as follows [36,37]:

(4) $$L(\left\{u_{k}\right\},\left\{\omega_{k}\right\},\lambda )=\alpha \sum_{k=1}^{K}{\left\|\partial_{t}\left[\left(\delta (t)+\frac{j}{\pi t})*u_{k}(t\right)\right]e^{-j\omega_{k}t}\right\|}_{2}^{2}+{\left\|f(t)-\sum_{k=1}^{K}u_{k}(t)\right\|}_{2}^{2}+\langle \lambda (t),f(t)-\sum_{k=1}^{K}u_{k}(t)\rangle$$

in which, α denotes the balancing parameter of the data-fidelity constraint. Equation (4) is then solved with the alternate direction method of multipliers [43]. All the modes gained from solutions in the spectral-domain are written as follows:

(5) $${\hat{u}}_{k}^{}(\omega )=\frac{\hat{f}(\omega )-\sum_{i\neq k}{\hat{u}}_{i}^{}(\omega )+\frac{\hat{\lambda }(\omega )}{2}}{1+2\alpha {\left(\omega -\omega_{k}^{}\right)}^{2}}$$

where the $\omega_{k}$ is computed at the gravity center of the power spectrum of the corresponding mode. Thus, Wiener filtering is embedded into the VMD algorithm to make it become more robust to sampling and noise.

(6) $$\omega_{k}^{}=\frac{\int_{0}^{\infty }\omega {\left|{\hat{u}}_{k}^{}\right|}^{2}d\omega }{\int_{0}^{\infty }{\left|{\hat{u}}_{k}^{}\right|}^{2}d\omega }$$

The detailed description of the complete VMD algorithm could be found in [36].

Applying the VMD algorithm to the received data after pre-processing, the animal VIMFs could be acquired, and according to the frequency range of breathing and heartbeat, the $u_{r}$ and $u_{h}$, which represent heartbeat components, could be extracted.

## 3. Results

### 3.1. Results of Dog

**Figure 4:**
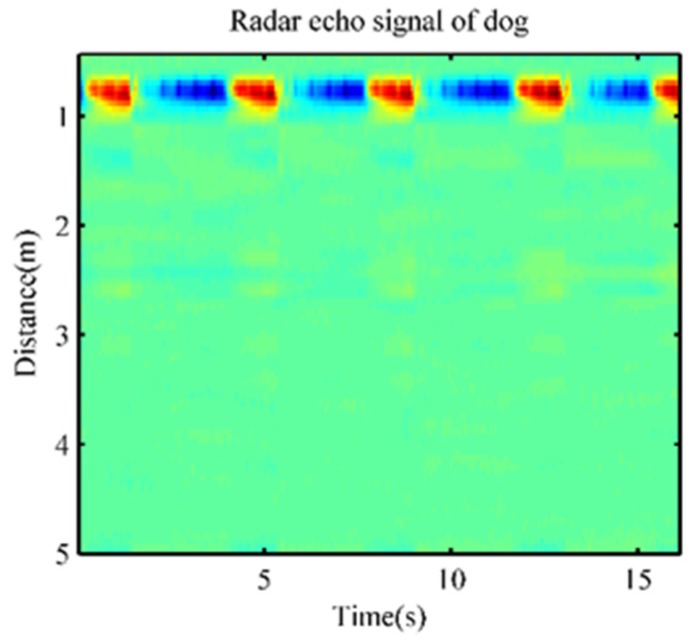
The radar echo signal of the dog.

As shown in Figure 3a, a beagle was used as a target. It was simultaneously measured by ECG and radar for physiological characteristics under anesthesia. The radar echo signal of the dog is shown in Figure 4, in which the distance information of the target and its periodic respiratory signal were obvious. The target could be easily located by calculating the energy at different distances.

**Figure 5:**
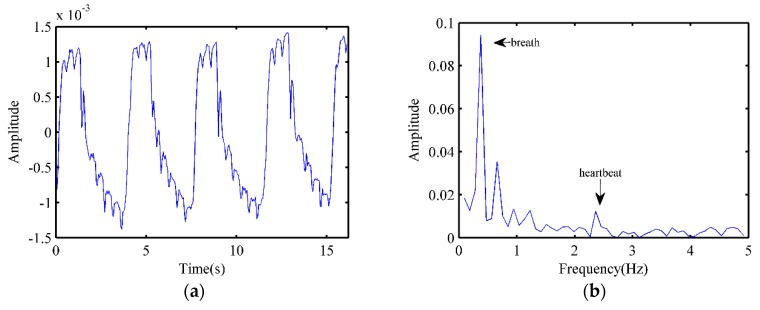

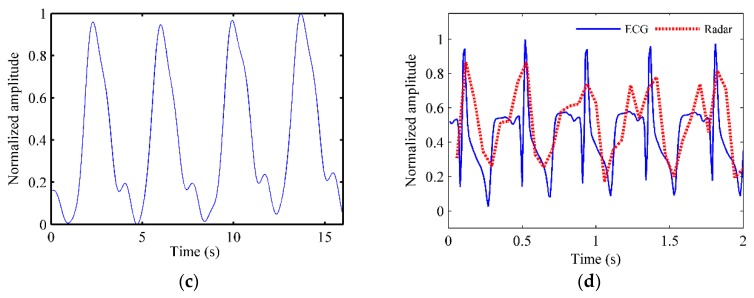
Experimental results. (**a**) The radar echo signal of the dog; (**b**) The spectrum of radar echo signal of the dog; (**c**) Breathing signal of the dog obtained by filtering; (**d**) Heartbeat signal received by radar and electrocardiogram (ECG), the inset shows waveform details at a selected 2-second duration.

After the background removal process, the waveform of the radar echo signal of the dog is shown in Figure 5a, where obvious respiratory waveforms and weaker heartbeat pulsations could be observed. By applying FFT, the signal spectrogram was obtained and shown in Figure 5b, by which, we could see that the respiratory rate of the dog could be directly obtained by FFT, and the heartbeat component was much weaker than respiration. Based on the frequency of breathing and heartbeat, high-pass and low-pass filtering were applied to the signals, respectively. Furthermore, waveforms of the respiratory and heartbeat are shown in Figure 5c,d, respectively. The cardiogram waveforms of ECG and radar in Figure 5d rendered very similar beat-to-beat intervals, while the radar feature points compared to the ECGs and T feature points would need further characterization. However, the radar was sufficiently accurate to replace ECG for behavior studies based on heart rate variation.

Although the heartbeat signals could be conveniently obtained using filters for known heartbeat frequencies, it is difficult to select an appropriate filtering range in practical applications. To accurately measure the heart rate, the VMD algorithm was used to extract heartbeat signals. An 8-second signal was selected for processing to better reflect changes in heart rate. Since the signal length of each processing was short and could only accommodate about two breathing cycles, there existed errors in calculating the breathing rate. Therefore, in this study, the VMD was only used to measure the heart rate.

The pre-processed data was given as the input parameters of the VMD algorithm. The number of modes was set to four. The balancing parameter and the tolerance were set to 10,000 and 10^−6^, respectively. Subsequently, the decomposition results could be obtained in real-time. According to the frequency range, VIMF1 was the component of the respiratory or low-frequency direct-current (DC), while VIMF2 was the heartbeat signal.

**Figure 6:**
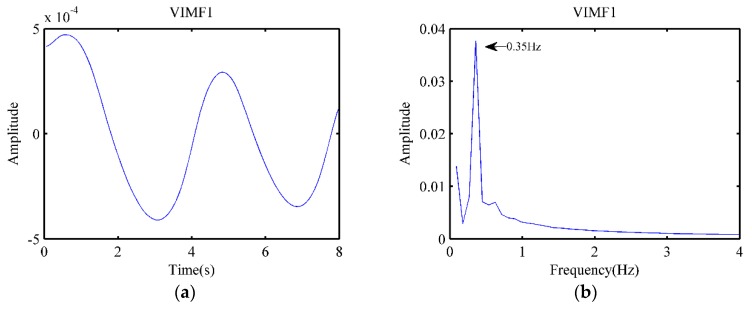

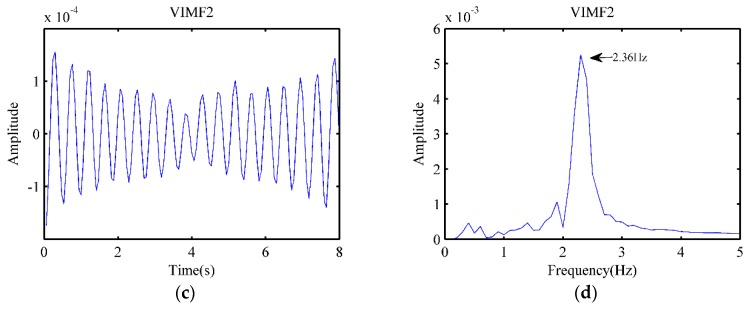
Results after the variational mode decomposition (VMD). (**a**) The waveform of VIMF1; (**b**) The spectrum of VIMF1; (**c**) The waveform of VIMF2; (**d**) The spectrum of VIMF2.

As shown in Figure 6a,b, only two breath cycles were included in the 8-second signal, while the respiratory frequency obtained by FFT was 0.35 Hz. It exhibited an error with the frequency calculated by the 16-second signal in Figure 6b, which was 0.378 Hz. The waveform of VIMF2 is shown in Figure 6c, while the spectrum of VIMF2 is shown in Figure 6d. We could see the regular heartbeat signal cycle in the figure. The heartbeat frequency calculated by FFT was 2.36 Hz, and it was close to the result in Figure 5b, which was 2.361 Hz. Meanwhile, the heartbeat frequency of the ECG signal corresponding to the segment sample was 2.397; the heart rate measured by the radar had only 1.54% error compared to ECG.

**Figure 7:**
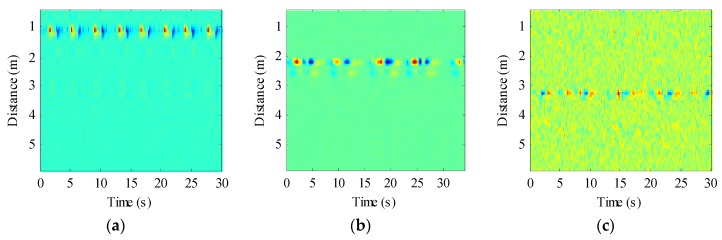
Received radar echoes after background removal. (**a**) Target is 1 m away from the radar; (**b**) Target is 2 m away from the radar; (**c**) Target is 3 m away from the radar.

**Figure 8:**
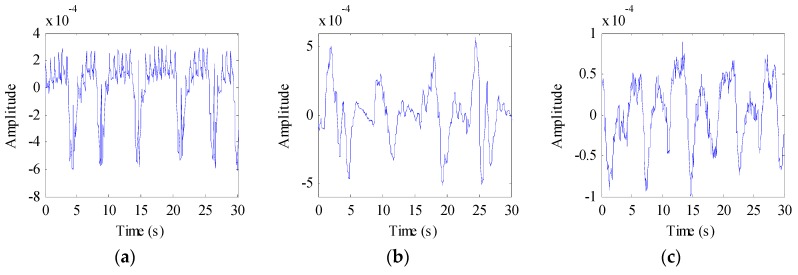
The waveform of target vital signs. (**a**) Target is 1 m away from the radar; (**b**) Target is 2 m away from the radar; (**c**) Target is 3 m away from the radar.

**Figure 9:**
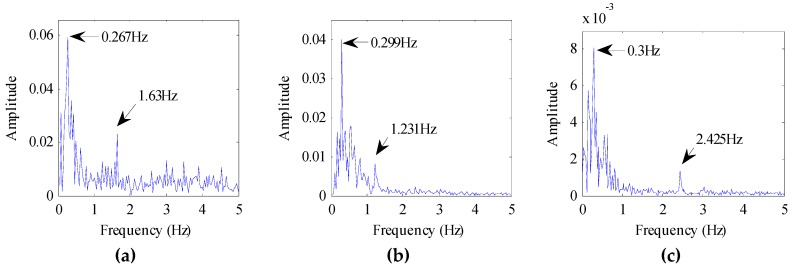
The spectrum of target vital signs. (**a**) Target is 1 m away from the radar; (**b**) Target is 2 m away from the radar; (**c**) Target is 3 m away from the radar.

**Figure 10:**
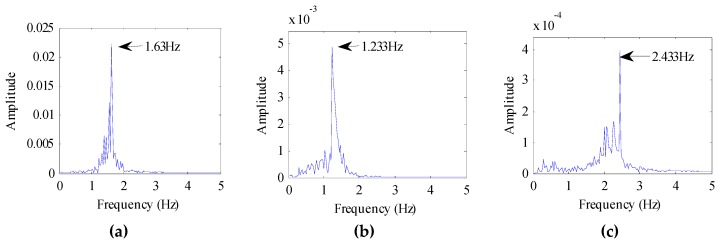
Frequency of target heartbeat. (**a**) Target is 1 m away from the radar; (**b**) Target is 2 m away from the radar; (**c**) Target is 3 m away from the radar.

We also evaluated the beagle dogs in an awake state (without anesthesia). They were placed on the test bench one by one, as shown in Figure 3b. The distance between the dogs and the radar was 1 m, 2 m, and 3 m, respectively. The echo data received by the radar is shown in Figure 7. The dog-radar distance was 1 m in Figure 7a, 2 m in Figure 7b, and 3 m in Figure 7c, respectively. In the figure, we could clearly see the target location. The selected vital sign signal of the target and its waveform is shown in Figure 8. By applying the FFT, we could directly calculate the respiratory rate of the target. The spectrum of target vital signs is shown in Figure 9. In the figure, we could see the respiratory frequency of the target corresponding to a different location was 0.3 Hz, 0.299 Hz, and 0.267 Hz. Compared to the measurement results of the contact sensor, the error was within 3%. Furthermore, the VMD algorithm was used to calculate the target heart rate, while the heartbeat frequency is shown in Figure 10. Because the experimental environment differed from the target daily life environment, the heart rate of the dog varied greatly during the measurement.

The vital signs of the three beagle dogs were measured at a distance of one meter from the radar. The obtained results are shown in Table 1. We could observe that the respiratory rate measured by the radar is consistent with the results of the pressure sensor, and the heart rate could also be obtained by the proposed method.

### 3.2. Experiment on Cat

**Figure 11:**
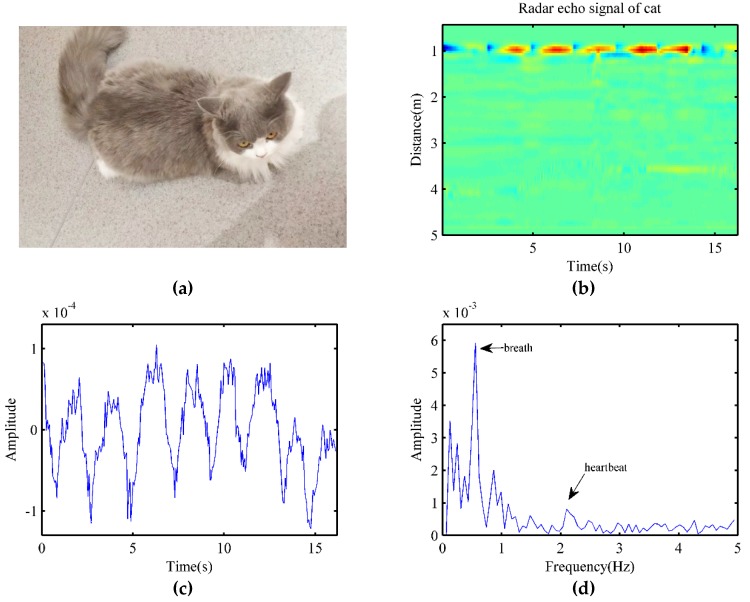
(**a**) A ragdoll cat that is used as a target in the experiment; (**b**) Radar echo signal of a cat; (**c**) The signal waveform of the cat received by radar; (**d**) The signal spectrum of the cat.

In this part, totally five cats participated in the experiment as detection objects. Figure 11a shows a Ragdoll cat as a subject. The radar was about 1 m away from the cat, which was in a calm state while being detected. After signal preprocessing, the radar echo signal is shown in Figure 11b. The target signal is very clear in the figure and could be easily located by calculating the maximum energy along the distance axis.

After the target location, the signal waveform of the cat is shown in Figure 11c, the breathing cycle of the cat was very clear, while the pulse waveform of the heartbeat was also visible. By applying an FFT, the signal spectrum of the cat is shown in Figure 11d. The breathing rate could be obtained directly from the spectrum, which was 0.556 Hz, and it was consistent with the results of the contact pressure sensor.Besides, the heartbeat frequency observed from the figure was 2.10 Hz. Meanwhile, we could see that the influence of animal hair on the measurement of vital signs in radar sensors was not significant. However, as the heartbeat signal was smaller than respiration, the VMD algorithm was applied to measure the heart rate of the cat.

**Figure 12:**
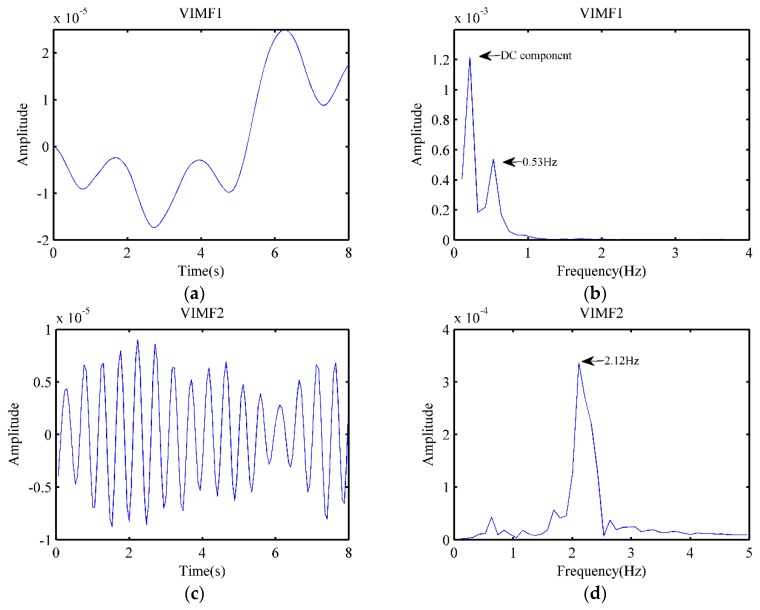
Results after VMD. (**a**) The waveform of VIMF1; (**b**) The spectrum of VIMF1; (**c**) The waveform of VIMF2; (**d**) The spectrum of VIMF2.

Similar to the settings in 3.1, the data after pre-processing was taken as the input of the VMD algorithm, and the parameters of VMD were the same as those for the dog. To better reflect changes in heart rate, the data taken as input was an 8-second signal, while the VIMFs obtained by the VMD algorithm are shown in Figure 12a,c, respectively. Meanwhile, after applying an FFT, the obtained spectrum of VIMF1 and VIMF2 are shown in Figure 12b,d, respectively. Compared to the dog, the cat exhibited higher breathing frequency, and there existed a low-frequency DC component in the signal, which prevented the respiratory amplitude in the VIMF1 spectrum from being significant enough. For heart rate measurement, the VIMF2 was our focusing point. In Figure 12c,d, we could claim that the heartbeat signal was well extracted, and the heart rate of the cat was well measured, while the heartbeat frequency was 2.12 Hz. Because the heart rate of a cat changes slightly with time, it was normal that the calculated heart rate differed from that in Figure 11d.

The vital signs of the five cats were measured at a distance between 1 m and 1.5 m away from the radar. The obtained results are shown in Table 2. We observed the respiratory rate measured by the radar had high accuracy compared to the pressure sensor, while the proposed method could also evaluate the heart rate of a cat.

## 4. Discussion

This study was the first to monitor the respiratory and heart rate of cats and dogs using a UWB radar. Radar was an unconstrained and non-contact measurement that would give the subject a more comfortable monitoring environment. It also avoided the stress on the subject that a contact sensor might cause, which would alter the physiological parameters of the subject. Compared to traditional methods (ECG, straps), the proposed method was not affected by animal hair; therefore, it did not require the removal of the target’s hair before measuring, which is very important for the welfare of animals.

Meanwhile, the proposed method was also a harmless measurement approach. The specific absorption rate (SAR) analysis of the radar sensor used in this study is given in Appendix A. The results revealed that the radar sensor used in this study met international standards and did little harm to the subjects.

We verified the accuracy of the respiratory rate measurement for cats and dogs by using a contact pressure sensor as a reference. In the experiment, the subjects were awake, rather than under anesthesia or recovering from anesthesia; the experimental results showed that the radar sensor had high measurement accuracy. Meanwhile, as a non-contact measurement device, radar had no restraint on the subjects and was less affected by animal hair, which made it useful for breathing monitoring during animal sleeping.

By comparing the obtained results with those of the ECG equipment, we verified the feasibility and accuracy of the radar measurement of heart rate. Due to the high accuracy of the radar sensor, we could not only obtain the heart rate from the echo signal but also more detailed information, such as the timing information of the P-wave, -waves, and T-wave carried in ECGs, which have been demonstrated and experimentally verified using human subjects in [44].

On the other hand, the shortcoming of the proposed scheme was its inability to measure vital signs for animals in motion. When the animal had a movement, such as running and jumping, it was difficult for the radar to measure the breathing and heart rate of the target because the breathing signal was very weak compared to the motion signal, and the measurements of breathing and heart rate during moving might require support from more advanced signal processing methods. Therefore, the practicality of the proposed method was more reflected in the vital signs monitoring of at-rest pets rest, such as sleep monitoring or measurement when animals cooperate.

Limited by experimental equipment, we were not able to fully confirm the accuracy of animal heart rate under other non-sedative conditions. Although the pressure sensor could measure the vital signs of animals, the data obtained also had limitations, in which the breathing component we extracted was strong, and the heartbeat component was weak. It was difficult to obtain an accurate heart rate as the ground truth for radar. Therefore, the results of the radar measurement of animal heart rate also need to be compared with ECG under non-sedated conditions, which would also be part of our future work.

With the development of radar technology, the cost of the UWB radar sensor is decreasing, which makes the proposed solution highly practical. Although we did not conduct experiments on wooden and plastic carriers, according to [45], it could be claimed that the electromagnetic waves in the frequency band we used have good penetration performance for these materials, and the penetration attenuation could be below 4 dB, usually around 0–3 dB. Besides, the radar sensor applied in this study is reported to successfully detect human vital signs through a wall in [46]. Therefore, it has the potential to penetrate pet cages and houses for vital signs monitoring, which would bring great convenience to the daily monitoring of cats and dogs.

## 5. Conclusions

As important members of the family, pets need to be monitored for their health status due to their limited communication abilities. In this study, we demonstrated a novel non-contact method to measure the vital signs of small cats and dogs. The UWB radar system with a convenient setup not only provided the previously unachievable sensing capability but also improved animal health monitoring programs with no harm to their welfare or interference with their circadian rhythms.

To verify the feasibility and accuracy of non-contact vital signs detection via UWB radar sensors, we conducted experiments on the pets. Using the bandage pressure sensor as a reference, we measured the breathing of the animal using a radar sensor while it was resting, and the experimental results demonstrated the performance of the proposed method. Besides, we also measured the heart rate of anesthetized animals using radar and compared the results with ECG to verify the feasibility of heart rate measurement. The experimental results demonstrated that the approach could meet the needs of monitoring pet breathing, and it also had the potential for the animal’s heart rate monitoring.

In the future, detection for other pets could be investigated due to the advantages of the UWB radar signal. Moreover, realizing the vital sign measurement of moving targets through a radar sensor would also be one of the further research directions.

## Figures and Tables

**Table 1:** Vital sign measurement results of three beagle dogs.

Subject	Breathing (Hz)		Error	Heartbeat (Hz)
	Radar	Pressure Sensor		
S1	0.266	0.27	1.5%	1.63
S2	0.412	0.42	1.9%	2.10
S3	0.219	0.23	4.8%	1.83

**Table 2:** Vital sign measurement results of five cats.

Subject	Breathing (Hz)		Error Rate	Heartbeat (Hz)
	Radar	Pressure Sensor		
S1	0.556	0.56	0.7%	2.10
S2	0.447	0.46	2.9%	2.68
S3	0.497	0.49	1.4%	2.88
S4	0.547	0.56	2.2%	1.99
S5	0.483	0.47	2.8%	2.17

## Appendix A

The International Commission on Non-Ionizing Radiation Protection (ICNIRP) released guidelines on electromagnetic safety in 1998 [47] and reconfirmed the document in 2012. ICNIRP defines SAR limits for electromagnetic fields, to prevent whole-body heat stress and excessive localized tissue heating. SAR is defined as the power absorbed per mass of tissue, with the units of watts per kilogram (W/kg). ICNIRP states that the reference levels were developed with the safety of extra sensitive population groups, such as elderly, infants, and younger children, in mind. The following basic restrictions apply in the frequency range from 100 kHz to 10 GHz.

ICNIRP has defined reference levels for comparison with measured values of physical quantities. Compliance with all reference levels given in these guidelines would ensure compliance with the basic restrictions given in Table A1. The relevant reference levels are:

**Table A1:** SAR (specific absorption rate) basic restrictions.

	Whole-Body Average SAR (W/kg)	Localized SAR (Head and trunk) (W/kg)	Localized SAR (Limbs) (W/kg)
SAR limit, general public	0.08	2	4

A note for pulsed systems suggests that “Seq (power density) as averaged over the pulse width should not exceed 1000 times the reference levels or that field strengths should not exceed 32 times the field strength reference levels”. The reference levels are given for the condition of maximum coupling of the electrical field to the exposed individual and, therefore, provide maximum protection.

The electrical and magnetic fields, *E* and *H* fields, could be calculated from the power density. Power density, at a given distance R, could be calculated by using the following formula:

(7) $$S=\frac{EIRP}{4\pi *R^{2}}$$

where EIRP is the transmitted power. ICNIRP defines the far-field as the region where the distance from a radiating antenna exceeds the wavelength of the radiated electric, magnetic, and electromagnetic fields. For objects in the far-field, the electrical field strength is then given by

(8) $$E=\sqrt{S\cdot Z_{0}}$$

where Z0 is the characteristic impedance of vacuum, $Z_{0}=120\pi \Omega$.

The magnetic field strength is given by

(9) $$H=E/Z_{0}$$

The magnetic flux density and the magnetic field strength are proportionally dependent when propagating in air. Thus, in describing a magnetic field for protection purposes, only one of the quantities *H* or *B* needs to be specified.

A device emitting energy in the 2–300 GHz range complies with the SAR limits if the field strengths are less than listed in Table A2. The power consumption of this radar system is 118.1 mW. The electrical field calculated from Equation (8) is 4.70 V/m; it is 13 times less than the ICNIRP reference level.

**Table A2:** ICNIRP reference levels for public exposure.

Frequency Range	E-field Strength (V/m)	H-field Strength (A/m)	B-Field (QT)	Equivelant Plane Wave Power Density S_eq_ (w/m2)
2–300 GHz	61	0.61	0.20	10
